# Differential Contributions of Fibroblast Subpopulations to Intercellular Communication in Eosinophilic Esophagitis

**DOI:** 10.3390/biology13070461

**Published:** 2024-06-21

**Authors:** Tao Li, Matthew Salomon, Ling Shao, Atousa Khalatbari, Joshua D. Castle, Anisa Shaker

**Affiliations:** 1 Swallowing and Esophageal Disorders Center, Division of Gastrointestinal and Liver Diseases, Department of Medicine, Keck School of Medicine of USC, Los Angeles, CA 90089, USA; 2 Research Center for Liver Diseases, Keck School of Medicine of USC, Los Angeles, CA 90089, USA; 3 Independent Researcher, Los Angeles, CA 90089, USA

**Keywords:** cell–cell communication, eosinophilic esophagitis, fibroblasts, fibroblast sub-populations, pathway analysis, single-cell RNA sequencing data analysis

## Abstract

**Simple Summary:**

Eosinophilic esophagitis is a chronic disease that can be complicated by fibrosis and strictures in the esophagus. Fibroblasts are a cell type that are considered important mediators of this process. The aim of this study was to better understand how fibroblasts can cause strictures by using publicly available single-cell RNA sequencing data to identify the different types of fibroblasts in patients with eosinophilic esophagitis. We identified at least two types of fibroblasts in eosinophilic esophagitis. Our analysis shows that fibroblasts are important contributors to the communication that occurs between cells in the human esophagus and that each of the fibroblast types has a unique way of interacting with other cell types (e.g., with epithelial cells or immune cells). This work can be used to provide additional insights into how strictures form in eosinophilic esophagitis.

**Abstract:**

Fibroblast heterogeneity remains undefined in eosinophilic esophagitis (EoE), an allergic inflammatory disorder complicated by fibrosis. We utilized publicly available single-cell RNA sequencing data (GSE201153) of EoE esophageal biopsies to identify fibroblast sub-populations, related transcriptomes, disease status-specific pathways and cell–cell interactions. IL13-treated fibroblast cultures were used to model active disease. At least 2 fibroblast populations were identified, F_A and F_B. Several genes including *ACTA2* were more enriched in F_A. F_B percentage was greater than F_A and epithelial–mesenchymal transition upregulated in F_B vs. F_A in active and remission EoE. Epithelial–mesenchymal transition was also upregulated in F_B in active vs. remission EoE and TNF-α signaling via NFKB was downregulated in F_A. IL-13 treatment upregulated ECM-related genes more profoundly in ACTA2− fibroblasts than ACTA2+ myofibroblasts. After proliferating epithelial cells, F_B and F_A contributed most to cell–cell communication networks. ECM–Receptor interaction strength was stronger than secreted or cell–cell contact signaling in active vs. remission EoE and significant ligand–receptor pairs were driven mostly by F_B. This unbiased analysis identifies at least 2 fibroblast sub-populations in EoE in vivo, distinguished in part by *ACTA2*. Fibroblasts play a critical role in cell–cell interactions in EoE, most profoundly via ECM–receptor signaling via the F_B sub-group.

## 1. Introduction

Eosinophilic esophagitis (EoE) is a Th2 immune/antigen-mediated clinicopathologic disorder characterized by symptoms of esophageal dysfunction and an esophageal, eosinophil predominant inflammation of ≥15 eosinophils/hpf in mucosal biopsies. Worsening dysphagia and esophageal food impactions requiring endoscopic interventions and the need for esophageal dilations are the most significant complications of EoE. The prevailing theory is that chronic, untreated and/or persistent inflammation (e.g., due to delays in diagnosis or therapy) can initiate a tissue remodeling process in the esophagus that involves epithelial hyperplasia, subepithelial fibrosis, and hypertrophy of the esophageal smooth muscle, that progresses, in many, though not all adults, to a fibrostenotic phenotype with strictures and luminal narrowing [1,2,3,4,5]. 

The pathogenesis of fibrostenosis in EoE is an active area of investigation and remains incompletely defined [6]. Activated fibroblasts are considered one of the primary effector cells in fibrosis, via deposition of connective tissue components including extracellular matrix (ECM) structural and adhesive proteins such as collagen and fibronectin and ground substance [7,8]. In EoE, cell-culture-based studies have shown that pro-fibrotic fibroblast behavior is regulated by Th2 cytokines prevalent in EoE (IL-4, IL-13, IL-5) and TGFβ1 secreted from a number of cells including eosinophils [9]. Pro-fibrotic fibroblast behavior has also been shown to be regulated by crosstalk with the extracellular matrix [10], endothelial [11], and epithelial [12] cells. Finally, epithelial cells have been shown to acquire fibroblast characteristics through epithelial–mesenchymal transition [13]. 

It is becoming increasingly clear, however, that fibroblasts are a heterogeneous population that remains incompletely defined in the human esophagus. Single-cell surveys of other organs and disease states such as the human colon and inflammatory bowel disease have revealed heterogeneity of stromal cell populations. Myofibroblasts, pericytes and 4 additional distinct populations of fibroblast-like cells have been identified in the healthy colon with shifts in these populations in active colonic inflammation [14]. More recently, single-cell RNA profiling of the human esophagus has revealed the existence of three major classes of stromal cells in the normal human esophagus (early immune, desmoplastic/ECM-depositing, and contractile fibroblast phenotypes) with shifts in these populations in the setting of Barrett’s esophagus, an intestinal metaplasia of the otherwise squamous epithelium [15]. 

There is also emerging evidence that fibroblast populations change with disease state in EoE with a recent study showing shifts in fibroblast populations in response to a TNF-related cytokine LIGHT. Spatial transcriptomics demonstrates an increase in LIGHT-regulated inflammatory fibroblasts in the epithelium in active EoE and a decrease in homeostatic fibroblasts in the lamina propria [16,17]. Despite these advances, the molecular features, heterogeneity, and cellular interactions of fibroblasts in the complex fibroinflammatory milieu of EoE remain incompletely defined.

Our aim was to leverage a publicly available dataset of single-cell RNA seq (scRNA-seq) of EoE patients [18] to identify fibroblast sub-populations, and related transcriptomes, disease status-specific pathways and cell–cell interactions in active and remission states. Active EoE was defined by histologic findings of ≥15 eosinophils per high-power field in esophageal biopsies and clinical symptoms. EoE in remission was defined by a history of EoE and eosinophils < 2 eosinophils per high power field in esophageal biopsies [18]. Herein we identify at least two fibroblast sub-populations in active and remission EoE, F_B and F_A, distinguished in part by *ACTA2*. The percentage of F_B is greater than F_A in active and remission EoE and characterized by genes related to epithelial–mesenchymal transition. TNF-α signaling via NFKB is downregulated in F_A and epithelial–mesenchymal transition is upregulated in F_B in active vs. remission EoE, respectively. Cell–cell communication analysis shows that fibroblasts play a critical role in cell–cell interactions in EoE, most profoundly via ECM-receptor signaling via the F_B sub-group.

## 2. Materials and Methods

### 2.1. Data Sources

Raw scRNA-seq data were extracted from Gene Expression Omnibus (GEO) under the accession number: GSE201153. 

### 2.2. scRNA-Seq Data Analysis

The scRNA-seq data were pre-processed for filtration, normalization and scaling using R toolkit Seurat (v4.1.2) [19]. After the selection and filtration of cells (genes expressed in at least 3 cells, cells with more than 200 genes represented by at least one UMI (unique molecular identifier) and percentage of counts coming from mitochondrial genes less than or equal to 5%), data normalization and scaling were performed using default options. Data were then batch corrected using Seurat’s integration method by all 10 samples, i.e., control (*n* = 2), EoE remission (*n* = 3), and active EoE (*n* = 5), to unbiasedly compare the cellular population repertoire. The datasets were first normalized with SCTransform. Seurat’s PrepSCTIntegration function was then applied prior to identifying anchors. In Seurat’s FindIntegrationAnchors and IntegrateData function, the normalization.method parameter was set to ‘SCT’. The top 20 principal components of the processed expression data were used to identify clusters based on Elbow plots (Supplementary Figure S1A). UMAP algorithm was used for dimensionality reduction. All dimensions of the low-dimension representation were used as input to generate the UMAP plots with default parameters. 

Marker genes were identified by determining the average log-fold change (logFC) of expression of each cluster compared to the rest of the cells using Seurat’s FindAllMarkers function using default settings for the Wilcoxon rank sum and MAST tests, which yielded the same commonly highly expressed genes in each cluster. We identified marker genes as those with an average log-fold change above 0.25, as previously described [18]. Clusters were labeled using cell types associated with the previously identified marker genes. The listed marker genes include, e.g., *CNFN* labeling epithelial cells, *GNLY* labeling lymphocytes, *LYZ* labeling myeloid, *TPSAB1* labeling mast cells, *PLVAP* labeling endothelial cells and *LUM* labeling fibroblasts. The acquired epithelial cell populations were also clustered into six subpopulations using Seurat with default settings, as previously described to annotate the epithelial subpopulations [18]. Listed marker genes included *KRT15* for Quiescent, *TOP2A* for Proliferating, *DSP* for Transitioning 1, *SERPINB3* for Transitioning 2, *TGM3* for Differentiated_low and *RNASE7* for labeling Differentiated_high, respectively.

### 2.3. Identification of Fibroblast Subpopulations

Data were batch corrected using Seurat’s integration method by 8 samples (i.e., EoE remission, and active EoE) as described above. Following integration, UMAP dimensionality reduction was performed on the first 20 principal components, again based on Elbow plot (Supplementary Figure S1B), resulting in 383 and 243 of the total number of fibroblasts in EoE remission and active EoE samples, respectively. Next, the integrated fibroblast population was further clustered into two and four subpopulations using Seurat with resolution set at 0.2 and 0.6 (determined by clustering tree using the R packages ‘clustree’ Seurat: v4.2.0 to ensure stable cluster), respectively, on the first 15 principal components (Supplementary Figure S1C). We did not perform sub-clustering in the control samples because there were only 26 fibroblasts. In the calculator from SATIJA lab that developed R package ‘Seurat’ v4.2.0, access date 20 September 2023 (https://satijalab.org/howmanycells/, accessed on 20 September 2023), we set the assumed number of cell types to 2 or 4, minimum fraction to 10% (we assumed there were no rare cell types) and minimum desired cells per type to 10 under the condition of 95% confidence. We found that if the cell number reached 167 or 179, at least 10 cells from each cluster could be accurately detected. The UMAP algorithm was used for dimensionality reduction.

Marker genes across two fibroblast subpopulations were identified using Seurat’s FindMarkers function with default settings with both Wilcoxon rank sum and MAST tests. A heatmap depicting the top 30 marker genes in each cluster was generated, where the listed gene order was based on adjusted *p*-value (<1 × 10^−10^). The lowest fold change among the marker genes was 1.6. 

### 2.4. DEGs and GSEA/Hallmark Database

Marker genes or differentially expressed genes across sub-clusters in active and in remission EoE and across disease status for each sub-cluster were identified with Seurat’s FindMarkers function using the default settings for both Wilcoxon rank sum and MAST tests, as described above, with similar results for each sub-cluster. Differentially expressed genes were considered with an FDR-adjusted *p* value threshold of q < 0.05 and a fold-change (FC) threshold of FC > 1.5. The whole gene lists identified with Seurat’s FindMarkers function using the default settings were used as inputs of R package ‘fgsea’ v1.30.0 with default parameters for gene set enrichment analysis (GSEA). Human hallmark functional collection was chosen as a resource of the GSEA molecular signatures database. Only pathways with an FDR-adjusted *p* value threshold of q < 0.25 were considered.

### 2.5. Cell–Cell Interaction

Cell–cell interaction was calculated using the R package ‘CellChat’ v1.5.0 and CellChatDB database with default parameters [20], only in EoE remission and active EoE samples. The above-mentioned Seurat outputs from these 8 samples with a resolution of 0.2 for fibroblasts sub-clustering, with the fibroblast population replaced by F_A and F_B sub-clusters, were used as the inputs. CellChatDB is generated based on the KEGG signaling pathway database and recent peer-reviewed studies and consists of three categories: secreted (paracrine/autocrine) signaling interactions, extracellular matrix (ECM)–receptor interactions, and cell–cell contact interactions.

The difference of incoming or outgoing interaction strength between all cell populations in active EoE and EoE remission via secreted signaling, ECM–receptor or cell–cell contact was obtained from CellChat, all of which were used as inputs of principal component analysis (PCA), performed by R packages ‘FactoMineR’ v2.9 and ‘factoextra’ v1.0.7 with default parameters. The total contribution scores of individual cell populations were calculated based on the scores from the first two dimensions of the PCA (Dimension 1 = 65.4% and Dimension 2 = 26.1%), following the equation: score in Dimension 1 × % Dimension 1 + score in Dimension 2 × % Dimension 2, which was used as inputs of R package ‘pheatmap’ to generate a heatmap. Significant ligand–receptor gene pairs (*p* < 0.05) between active and remission EoE, focused on the fibroblast sub-populations, were identified using the “statistically robust mean method” in the CellChat algorithm. The ligand–receptor pairs were classified into secreted, cell–cell contact, and ECM–receptor cell–cell type interactions. The communication probability between cell types was modeled by the law of mass action as described by Jin et al. [20]. 

### 2.6. Cell Culture and Treatment

Previously characterized primary human esophageal fibroblast/myofibroblast cultures, established from surgical resections of deidentified normal human esophagi were cultured as previously described [21,22]. These primary cultures have been previously shown to express *ACTA2+* gene and α-SMA protein. Human esophageal primary fibroblasts were purchased from ScienCell Research Laboratories (Carlsbad, CA, USA, Catalog no. 2730), and cultured in Fibroblast Medium (FM, Cat. #2301) as recommended by the manufacturer. To model active EoE, *ACTA2+* and *ACTA2−* fibroblasts (passage 2–6) grown in 6 well plates were treated with IL-13 (final concentration 100 ng/mL, Peprotech, Rocky Hill, NJ, USA, catalog no. 200-13) for 24 h, as previously described [18,23]. 

### 2.7. Real-Time Quantitative RT-PCR

RNA was isolated using the RNAeasy mini kit (QIAGEN, Germantown, MD, USA), followed by cDNA synthesis using SuperScript™ (Invitrogen™, Waltham, MA, USA) reverse transcription system according to the manufacturer’s protocol. Quantitative PCR (qPCR) was performed with the isolated cDNAs using SYBR Green Master Mix for *ACTA2*, *POSTN*, *LUM*, *RGS5*, *EGFL6*, *CCL26*, *TNC*, *IL13RA1*, *IL13RA2*, *FN1*, *COL6A1*, *COL6A2*, *COL6A3*, and *COL1A2* (Supplementary Table S1). Ten microliters of reaction medium contained 2 µL of diluted cDNAs and 8 µL of 300 nM gene-specific primers and SYBR Green Master Mix (Applied Biosystems, Waltham, MA, USA). A control analysis was performed with an RNA sample to detect contamination by genomic DNA and with water to exclude the formation of primer–dimers. The PCR program was conducted as follows on a QuantStudio 5 (Life Technologies, Carlsbad, CA, USA): one cycle at 95 °C for 2 min, and 40 cycles of 15 s at 95 °C and 1 min at 60 °C. Data collection was performed at 60 °C. The melting curve was produced according to the following program: 15 s at 95 °C at a ramp rate of 1.6 °C s^−1^, 1 min at 60 °C at a ramp rate of 1.6 °C s^−1^, heating to 95 °C at a ramp rate of 0.05 °C s^−1^ and held in place for 15 s. The data were continuously collected. The data were analyzed by the 2^−∆∆Ct^ method, using GAPDH and untreated conditions as references. qPCR was conducted in triplicate using at least three independent cDNAs.

### 2.8. Statistical Analysis 

At least three biological replicates for in vitro experiments and numerical data are presented as mean ± SD. The statistical analysis was performed using a 2-tailed Student’s *t*-test in Excel. We required replication at *p* < 0.05 using R packages v4.1.2. R scripts used for data analyses (Seurat, CellChat, fgsea, Monocle 3) in our study can be found in the public GitHub repositories (Supplementary Table S2). 

## 3. Results

### 3.1. Fibroblast Sub-Clusters in Total Dataset

**Figure 1:**
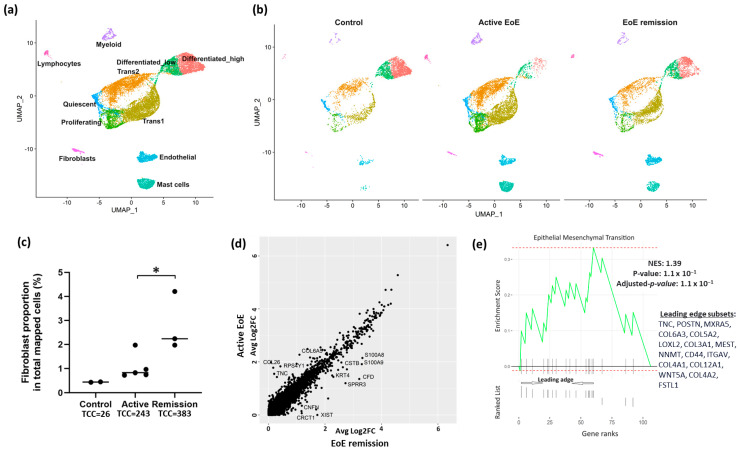
UMAP visualization of the clustering of cells from healthy controls, EoE remission and active EoE. Seurat’s integration method was used for batch-correction across control, EoE remission and active EoE datasets. (**a**) Labeled cell clusters, representing the combination of control, remission, and active EoE datasets. Six major cell types exist in control, EoE remission and active EoE: epithelial cells, lymphocytes, myeloid cells, mast cells, endothelial cells, and fibroblasts. Epithelial cells were further categorized into six subpopulations: Quiescent, Proliferating, Transitioning 1 (Trans1), Transitioning 2 (Trans2), Differentiated_low and Differentiated_high. (**b**) Separated cell clusters shown by disease status. (**c**) Proportion of fibroblasts in total mapped cells in normal (0.44%), active (1.05%), and remission (2.81%) EoE (active vs. remission, * *p* = 0.026 by 2-tailed Student’s *t* test) is shown. Total fibroblast cell count (TCC) is listed for each condition. (**d**) Differentially expressed genes (DEGs) in fibroblasts in active vs. remission EoE. The y-axis and x-axis show average log2FC in active vs. remission EoE and remission vs. active EoE, respectively, for the integrated fibroblast dataset. Individual dots reflect genes and annotated dots are DEGs with an absolute FC > 1.5 or log2FC of 0.6 and an FDR-adjusted *p*-value < 0.05. Genes along the diagonal line were unchanged and not annotated. (**e**) Gene Set Enrichment Analysis (GSEA) showing pathway enrichment changes in total fibroblasts in active EoE vs. EoE remission. All fibroblast marker genes in active vs. remission EoE were ranked by log2FC and used as inputs for GSEA. Dotted red lines show the minimum and maximum enrichment scores. Leading edges are shown and component genes of the leading edge subset are listed in order of rank. NES, normalized enrichment score. Positive NES represents the upregulation in active EoE.

Uniform manifold approximation and projection (UMAP) visualization of the clustering of all cells from GSE201153 confirmed the presence of 6 major cell clusters (Figure 1a). The top ten representative marker genes utilized by the original analysis of GSE201153 to identify cell types were confirmed in our re-analysis (Supplementary Tables S3–S5). Separation of cell clusters by disease status (Figure 1b) (control, active EoE, and EoE remission) confirmed that the proportion of fibroblasts in the total number of mapped cells was higher in remission versus active EoE (2.81% versus 1.05%, remission vs. active, *p* = 0.026) (Figure 1c). The low number of fibroblasts in the control sample (*n* = 26, 0.44%) prohibited further analysis in this group and additional analyses of the fibroblast populations were limited to the datasets from active EoE and remission EoE. There were 13 differentially expressed genes (DEGs) (Fold change > 1.5, FDR adjusted *p*-value < 0.05) in all fibroblasts in active versus remission EoE, including upregulation of *CCL26* and *TNC* in active disease (Figure 1d). Pathway enrichment based on GSEA of all marker genes and human Hallmark functional collection (FDR adjusted *p*-value < 0.25) identified upregulation of epithelial–mesenchymal transition in fibroblasts with the top leading edge genes, *TNC* and *POSTN*, contributing most to the enrichment scores (Figure 1e).

**Figure 2:**
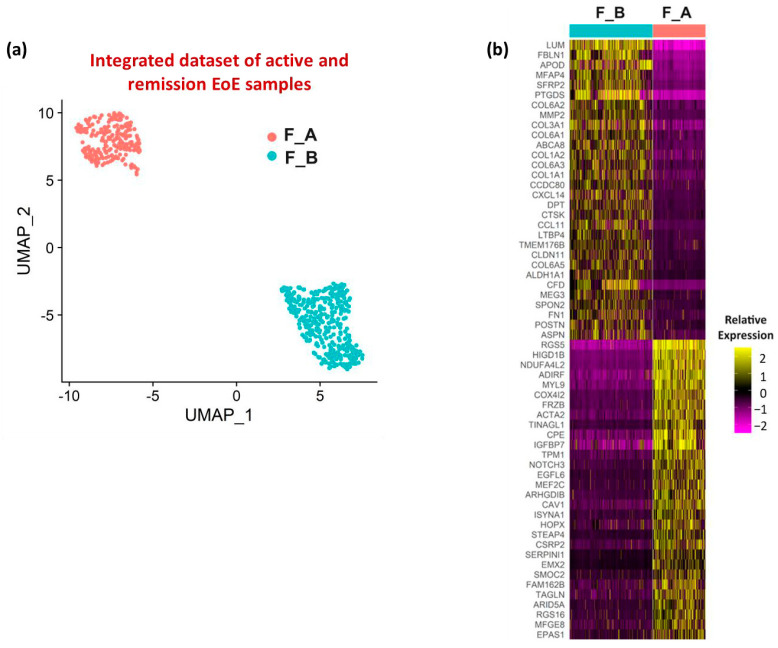
Fibroblast sub-clusters and associated enriched genes. (**a**) UMAP visualization of the fibroblast sub-clustering in the integrated dataset (active EoE and EoE remission) with resolution set at 0.2. (**b**) Heatmap depicting the relative expression levels based on average log2FC of the top 30 marker genes in each fibroblast sub-cluster in integrated active EoE and EoE remission datasets. Gene order is based on adjusted *p*-value (all < 1 × 10^−10^). The lowest FC among marker genes was 1.6.

To further define the fibroblast populations in EoE, UMAP visualization of fibroblast sub-clustering of integrated datasets from active and remission EoE was performed with a resolution set to 0.2 (resolution determined by clustering tree using the R packages ‘clustree’) and identified 2 fibroblast subpopulations (termed F_A and F_B) (Figure 2a). There was differential expression of the top ten representative fibroblast marker genes utilized by the original analysis of GSE201153 in F_B vs. F_A (Supplementary Table S5). Heatmap of the top 30 genes within each subgroup (order based on FDR adjusted *p*-value, color is based on average log2FC) (Figure 2b) showed *LUM* and *RGS5* were the top genes in F_B and F_A, respectively. Additional genes enriched in F_B were the elastic fiber genes *FBLN1* and *FN1* and several collagens (COLs), including *COL1A1*, *COL1A2*, *COL3A1*, *COL6A1*, *COL6A2*, *COL6A3*, and *COL6A5*. Additional and similarly enriched genes in F_A were contractile genes *MYL9*, and the TGFβ inducible contractile genes *ACTA2* and *TAGLN*, widely considered “myofibroblast” markers. 

### 3.2. Fibroblast Subclusters in Active EoE and in Remission EoE

**Figure 3:**
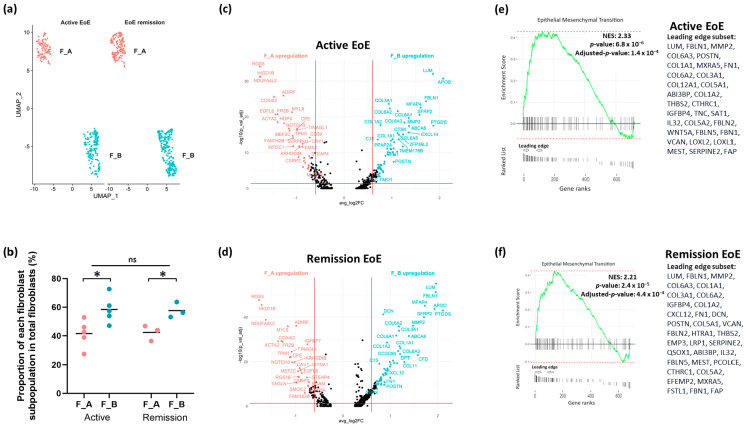
Fibroblast sub-clusters in active and remission EoE. (**a**) UMAP visualization of the fibroblast sub-clustering in active EoE and in EoE remission after integration with 0.2 resolution. The X-axis is duplicated from −10 to 5 to show active and EoE remission on the same graph. (**b**) The % of each fibroblast sub-population F_A or F_B in active EoE samples and in EoE remission samples (* *p* < 0.05 by 2-tailed Student’s *t* test, ns = not significant). (**c**) Volcano plot of top DEGs in active EoE in F_A vs. F_B. (**d**) Volcano plot of top DEGs in EoE remission in F_A vs. F_B. (**e**) GSEA derived top pathway from human Hallmark functional collection (FDR adjusted *p*-value < 0.25) is shown for F_B vs. F_A in active EoE (**e**) and in EoE remission (**f**). All fibroblast marker genes in F_A vs. F_B subpopulations in active EoE (**e**) and EoE remission (**f**) were ranked by log2FCand used as inputs for GSEA. Dotted lines show minimum and maximum enrichment scores. Leading edges are shown and the component genes of the leading edge subset are listed in order of rank. NES, normalized enrichment score. Positive NES represents upregulation of the pathway in F_B vs. F_A in active or remission EoE.

We were then interested in the distribution of these fibroblast sub-populations in active and remission EoE. UMAP of fibroblast sub-clusters by disease state mirrors the distribution of fibroblast sub-clusters in the integrated dataset (Figure 3a). The proportions of F_A and F_B fibroblast sub-populations remained similar in active EoE and remission EoE, with a greater % of F_B in total fibroblasts than F_A in both active (41.6% vs. 58.4%, *p* = 0.02) and remission EoE (42.3% vs. 57.7%, *p* = 0.02) (Figure 3b).

DEGs as determined per FindMarkers function in Seurat (FC > 1.5 and FDR adjusted *p*-value < 0.05) in F_B vs. F_A in each disease state showed that in active EoE, 88 and 63 genes were upregulated in the F_B and F_A sub-populations, respectively (Figure 3c). In remission EoE, 87 and 45 genes were activated in the F_B and F_A sub-populations, respectively (Figure 3d). Pathway enrichment based on GSEA of all marker genes and human Hallmark functional collection (FDR adjusted *p*-value < 0.25) showed upregulation of epithelial–mesenchymal transition in F_B vs. F_A in active EoE and in remission EoE, with the top leading edge gene *LUM* (Figure 3e,f). These results suggest that the upregulation of epithelial–mesenchymal transition in active vs. remission EoE observed in the integrated dataset is driven by the F_B sub-population. 

### 3.3. DEGs and Pathways in Fibroblast Sub-Clusters in Active versus Remission EoE

**Figure 4:**
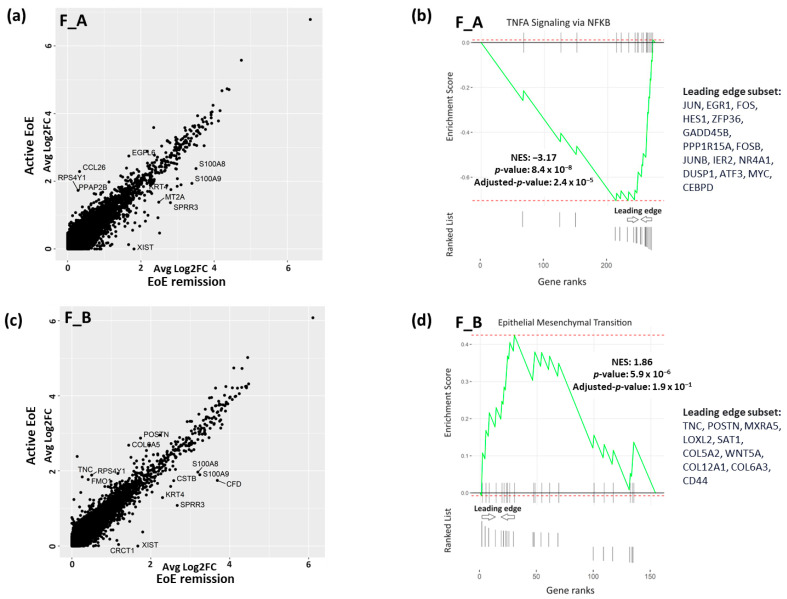
DEGs and pathway analysis in fibroblast sub-populations in active vs. remission EoE. (**a**,**c**) DEGs in F_A fibroblast (**a**) and in F_B fibroblast (**c**) subpopulations in active vs. remission EoE. Individual dots reflect genes and annotated dots are DEGs with an absolute FC > 1.5 or log2FC of 0.6 and an FDR-adjusted *p*-value < 0.05. y-axis and x-axis show average log2FC. Upregulated genes in active EoE are above the diagonal cluster and upregulated genes in remission EoE are below the diagonal cluster in the F_A (**a**) and F_B (**c**) subpopulations. (**b**,**d**) GSEA-derived top pathway from human Hallmark functional collection (FDR adjusted *p*-value < 0.25) is shown for F_A (**b**) and F_B (**d**) fibroblast sub-populations in active vs. remission EoE. Genes in F_A or F_B sub-populations in active vs. remission EoE were ranked by log2FC and used as inputs for GSEA. Dotted red lines show the minimum and maximum enrichment scores. Leading edges are shown and component genes of the leading edge subset are listed in order of rank. NES, normalized enrichment score. Negative or positive NES represent pathway downregulation or upregulation, respectively, in active vs. remission EoE for F_A (**b**) and F_B (**d**) fibroblast subpopulations.

We were also interested in whether there were transcriptomic differences in each sub-population in different disease states, i.e., active vs. remission EoE. For each subcluster (F_A and F_B) there were a set of DEGs as determined per FindMarkers function in Seurat (Fold change > 1.5, FDR adjusted *p*-value < 0.05), in active vs. remission EoE. There were 10 DEGs in F_A (Figure 4a) with CCL26 most uniquely upregulated (Fold change > 1.5, FDR adjusted *p*-value < 0.05) and 13 DEGs in F_B (Figure 4c) with TNC most uniquely upregulated (Fold change > 1.5, FDR adjusted *p*-value < 0.05) in active vs. remission EoE, respectively. Pathway enrichment based on GSEA of all marker genes and human Hallmark functional collection (FDR adjusted *p*-value < 0.25) showed downregulation of TNF-α signaling via NFKB in F_A in active vs. remission EoE with downregulation of the top leading edge genes *JUN*, *EGR1*, *FOS*, *HES1*, *ZFP36*, *GADD45B*, *PPP1R15A*, *FOSB*, *JUNB*, *IER2*, *NR4A1*, *DUSP1*, *ATF3*, *MYC*, and *CEBPD* (Figure 4b). Pathway enrichment analysis in F_B sub-cluster showed upregulation of epithelial–mesenchymal transition with upregulation of top leading genes *TNC*, *POSTN*, *MXRA5*, *LOXL2*, *SAT1*, *COL5A3*, *WNT5A*, *COL12A1*, *COL6A3*, and *CD44* in active versus remission EoE (Figure 4d). This finding further supports the contribution of F_B to epithelial–mesenchymal transition in EoE and also identifies a possible role for TNF via NFKβ in the F_A sub-cluster.

### 3.4. In Vitro Modeling of Active EoE in ACTA2+ and ACTA2− Fibroblast Cultures

**Figure 5:**
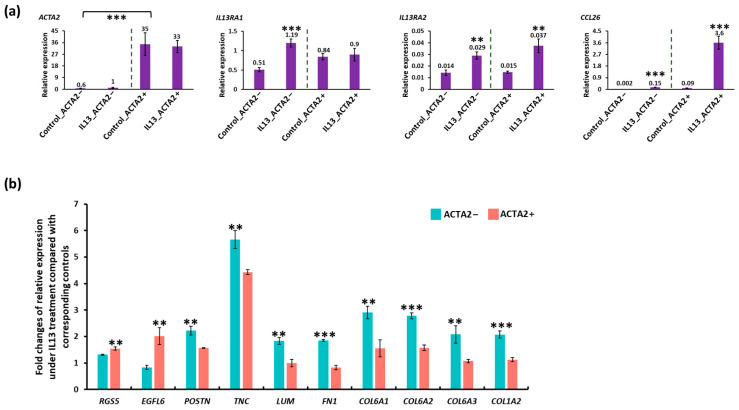
qRT-PCR of ACTA2+ and ACTA2− primary fibroblasts treated with IL13. (**a**) mRNA expression for each gene is shown relative to GAPDH in untreated and IL13-treated ACTA2− fibroblasts and ACTA2+ myofibroblasts. (**b**) Fold change in *RGS5*, *EGFL6*, *POSTN*, *TNC*, *LUM*, *COL6A1*, *2*, *3*, COL1A1 relative to untreated controls in ACTA2− fibroblasts and ACTA2+ myofibroblasts. ** *p* < 0.01, *** *p* < 0.001.

To begin to validate the scRNA seq, we utilized human primary fibroblast cultures with and without enrichment of ACTA2, one of the genes that distinguished the F_A and F_B subpopulations. We chose this approach because ACTA2 was identified by scRNA-seq as enriched in the F_A population and the ready availability of previously described α-SMA+ primary esophageal human myofibroblasts (termed *ACTA2+*) and commercially available primary human fibroblasts (termed *ACTA2−*). Active disease was modeled by treatment of *ACTA2+* and *ACTA2−* primary cultures with IL-13 (Figure 5). We confirmed stable enrichment of *ACTA2* expression in previously described human primary myofibroblast/fibroblast cultures compared to mRNA expression in purchased human primary fibroblasts in the presence and absence of IL-13. *IL13RA1* and *IL13RA2* expression were similar in untreated cells. In response to IL-13 treatment, *IL13RA1* expression increased only *ACTA2−* primary cultures and *IL13RA2* increased in both primary cultures. *CCL26* expression increased in both primary cultures confirming the efficacy of IL-13 treatment, although *CCL26* expression remained greatest in *ACTA2+* primary cells (Figure 5a). IL-13 treatment resulted in more profound upregulation of *RGS5* and *EGFL6* in *ACTA2+* cells, while *POSTN*, *TNC*, *LUM*, *FN1*, *COL6A1*, *COL6A2*, *COL6A3*, *and COL1A2* were more profoundly upregulated in *ACTA2−* cells (Figure 5b).

### 3.5. Cell–Cell Communication Analysis

Recognizing the complexity of the in vivo environment and the critical role of cell–cell interactions in homeostasis and disease, we utilized CellChat to interrogate the role of fibroblast subpopulations in relation to other cell types in EoE. CellChatDB is a comprehensive signaling molecule interaction database (consists of secreted signaling, ECM–receptor, and cell–cell contact signaling) included in the CellChat repository which is manually curated on the basis of the KEGG signaling pathway database and recently peer-reviewed publications. The whole dataset was used for this analysis including non-fibroblast cells. Among the validated interactions in the database, 61.8%, 21.7% and 16.5% of the interactions were involved in secreting, ECM–Receptor, and cell–cell contact signaling, respectively. 

**Figure 6:**
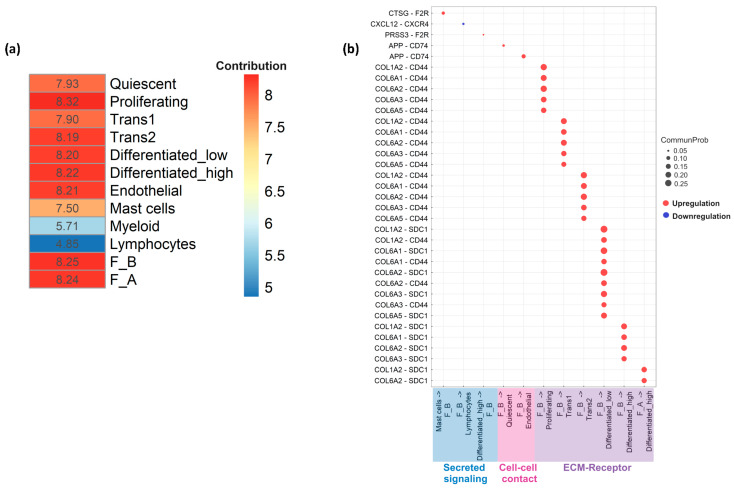
Cell–cell communication across all cell types in active EoE and remission EoE. (**a**) Heatmap summarizing the results from principal component analysis (PCA) of the contribution of all individual cell populations to EoE via secreted signaling, cell–cell contact, or ECM–receptor. The total contribution scores of individual cell populations were calculated based on the scores from the first two dimensions of the PCA (Dimension 1 = 65.4% and Dimension 2 = 26.1%), following the equation: score in Dimension 1 × % Dimension 1 + score in Dimension 2 × % Dimension 2. (**b**) Bubble plots show the significant ligand–receptor pairs involved in communication of F_A and F_B fibroblasts in active versus remission EoE and the other cell groups (lymphocytes, myeloid cells, mast cells, endothelial cells and epithelial subpopulations: Quiescent, Proliferating, Transitioning 1 (Trans1), Transitioning 2 (Trans2), Differentiated_low and Differentiated_high). Circle size represents the communication probability (interaction strength) with the communication probability ≥ 0.05 in secreted signaling and cell–cell contact and ≥0.15 in ECM–receptor. Red and blue bubbles indicate upregulation and downregulation in active vs. remission EoE, respectively. F2R, APP, COL1A2, COL6A1, COL6A2, COL6A3, COL6A5 are ligands and F2R is a receptor in fibroblasts.

Differences in incoming and outgoing signaling interactions amongst all cell populations in active EoE and EoE remission were used as inputs of principal component analysis (PCA). A heatmap reflecting the contribution of individual cell populations (based on a total score derived from the contribution to Dimension 1 (65.5%) and Dimension 2 (26.1%) of the PCA) showed proliferating epithelial cells, one of the epithelial sub-populations, contributed the most to variation in cell–cell communication networks in the samples (total contribution score 8.32), followed by the F_B (total contribution score 8.25) and F_A (total contribution score 8.24) fibroblast sub-populations (Figure 6a).

Of the 3 types of signaling, ECM–Receptor interaction strength (communication probability) was much stronger in active EoE vs. remission EoE while interaction strength for secreted and cell–cell contact signaling was similar in active and remission EoE. Significant ligand–receptor pairs and associated communication probabilities for each type of signaling, focused on fibroblast sub-populations, were then identified. ECM–receptor signaling consisted of multiple collagen sub-types from F_B population to 5 epithelial sub-populations (differentiated high, differentiated low, proliferating, trans1, and trans2). There was also a significant ligand–receptor pair from F_A to differentiated high epithelial cells via COL6A2-SDC1 and COL1A2-SDC1 (Figure 6b). Cell–cell contact signaling consisted of significant ligand–receptor pairs from F_B to endothelial and quiescent epithelial cells via APP-CD74 (Figure 6b). With secreted signaling, significant ligand–receptor pairs were detected from F_B to lymphocytes via CXCL12-CXCR4. F_B also received signals from mast cells and differentiated high epithelial cells via CTSG-F2R and PRSS3-F2R, respectively (Figure 6b). Although there was an overlap between the ligands identified for the F_A and F_B populations, significant ligand–receptor pairs were driven mostly by the F_B subpopulation (Figure 6b). 

### 3.6. Additional Subsets in the F_B Fibroblast Sub-Cluster

**Figure 7:**
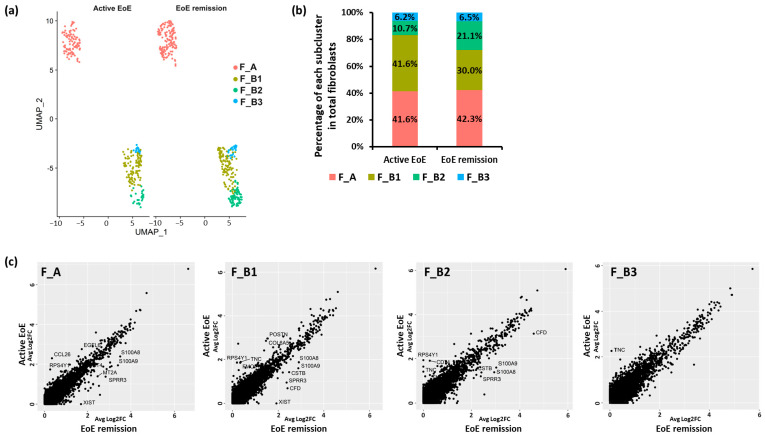
Fibroblast subclusters with resolution set at 0.6. (**a**) UMAP visualization of the fibroblast sub-clustering in active EoE and EoE remission after integration with resolution set at 0.6. The X-axis is duplicated from −10 to 5 in order to show active EoE and EoE remission on the same graph. (**b**) The percentage of each subcluster of fibroblasts in all fibroblasts is shown for active EoE and EoE remission. (**c**) Top DEGs in each fibroblast sub-cluster between active EoE and EoE remission. DEGs were determined with absolute fold changes > 1.5 and an FDR-adjusted *p*-value < 0.05.

To begin to determine if there were additional fibroblast subsets within F_A and F_B contributing to these cell–cell interactions, UMAP visualization of fibroblast sub-clustering of integrated datasets from active and remission EoE was performed with a resolution set to 0.6 (determined by a clustering tree using the R packages ‘clustree’, Supplementary Figure S2). Sub-clustering identified 3 subpopulations within F_B (F_B1, F_B2, F_B3) without additional F_A subpopulations (Figure 7a). There appeared to be a higher % of F_B1 in active versus remission EoE (41.6% vs. 30.0%), and a higher % of F_B2 in remission versus active EoE (10.7% vs. 21.1%), with a similar % of F_B3 in active versus remission (6.2% vs. 6.5%) (Figure 7b). 

For each subcluster (F_A, F_B1, F_B2, F_B3) there were a set of DEGs as determined per FindMarkers function in Seurat (Fold change > 1.5, FDR adjusted *p*-value < 0.05), between active EoE and EoE remission (Figure 7c). There were 10 DEGs in F_A, 12 DEGs in F_B1, 9 DEGs in F_B2, and 1 DEG in F_B3 in active versus remission EoE. CCL26 remained most upregulated in F_A (Fold change > 1.5, FDR adjusted *p*-value < 0.05) and TNC was most upregulated in F_B1, F_B2, and F_B3 (Fold change > 1.5, FDR adjusted *p*-value < 0.05) in active versus remission EoE. Upregulation of POSTN was also identified only in F_B1. Numbers in each of the F_B sub-populations were too small to perform additional analysis. 

## 4. Discussion

Our study supports the increasingly recognized role of a heterogeneous stroma in the pathogenesis of EoE. This unbiased analysis of publicly available single-cell RNA-Seq dataset of EoE patients identifies at least 2 fibroblast sub-populations in active and remission EoE in vivo, which we called F_B and F_A, and which were distinguished by enrichment for several markers including ACTA2. The F_B population accounted for a greater percentage of the fibroblast population than F_A in both active and remission EoE. F_B is further distinguished from F_A by gene expression related to epithelial–mesenchymal transition in both active and remission states. Pathway enrichment analysis also shows that in active versus remission EoE, TNF-α signaling via NFKB is downregulated in F_A and that epithelial–mesenchymal signaling is upregulated in F_B. Interestingly, the F_B subpopulation appears to have additional subsets with unique DEGs in the active versus remission states. In addition, there appear to be shifts in these additional subsets related to disease state. Finally, despite an overall low absolute number of fibroblasts compared to other cell types in the available samples, cell–cell communication analysis demonstrates that fibroblasts play a critical role in cell–cell interactions in EoE, most profoundly via ECM–receptor signaling via the F_B sub-group. In vitro modeling of active EoE with IL-13 treatment of fibroblast primary cultures, chosen to reflect some of the characteristics of the F_A and F_B populations, also shows differential upregulation of genes, supporting the findings from our analysis of this dataset.

Studies of the sub-epithelial space and stroma in the esophagus in normal or diseased states including EoE have been limited due to inherent difficulties associated with sampling [9]. Studies using esophageal biopsies from normal human esophagus with sufficient lamina propria for analysis, demonstrated that most cells in the sub-epithelial space were vimentin expressing non-immune, non-endothelial stromal cells termed fibroblasts. In addition, there were scattered α-SMA+ and vimentin+ non-endothelial, non-immune cells, subjacent to the epithelium which were termed myofibroblasts. In samples with histologic evidence of gastroesophageal reflux disease, there is an increase in these α-SMA+ and vimentin+ myofibroblasts compared to normal biopsies, although the percentage of myofibroblasts compared to fibroblasts, remained quite small [21]. A similar approach in EoE demonstrates an increase in immunofluorescent staining for activated fibroblasts or myofibroblasts in active disease that persists in the remission state in adult patients while biopsies from pediatric patients showed resolution of stromal activation [10]. Ex-vivo studies of fibroblasts isolated from patients with active EoE showed little baseline α-SMA expression, but an increase in response to TGFβ [16]. 

This study adds to the literature dissecting the multiple populations of stromal cells in the human esophagus. Single-cell RNA profiling of the human esophagus has revealed the existence of 7 clusters of stromal cells, encompassing three major classes in the normal human esophagus, with a decrease in the early immune PDGFRA^hi^ PDPN^hi^ CD34^hi^ class and an expansion in the desmoplastic/ECM-depositing PDGFRA^hi^ PDPN^hi^ CD34^lo^; and contractile classes in Barrett’s esophagus, compared to normal state [15]. In our work, small numbers of fibroblasts in the control samples from a publicly available database precluded the inclusion of the normal state in our analyses. However, we did not identify differential expression of PDGFRA, PDPN, or CD34 in the F_A or F_B sub-populations. Overall, based on DEG profiles, including enrichment for *ACTA2* and *MYL9*, the F_A population identified in our work has characteristics of the contractile phenotype identified in the earlier study. The F_B population seems similar to the previously identified desmoplastic/extracellular matrix-depositing phenotype characterized by the expression of several *COLs* and *POSTN*. 

Our work also adds to the existing literature suggesting the existence of multiple fibroblast subtypes in EoE with distinct roles. In EoE, an increase in expression of the TNF-related cytokine LIGHT has been previously reported. RNA in situ hybridization studies focused on esophageal fibroblasts’ response to the LIGHT, have demonstrated an increase in an inflammatory phenotype of fibroblast characterized by *VIM+ICAM-1+IL-34+* cells in active EoE compared to normal biopsies [16,17]. In our unbiased study of the publicly available scRNA-seq dataset of EoE patients, we did not identify changes in *ICAM1* and *IL34* expression in F_A or F_B populations in our analysis. We also show that although the % of the F_A population is less than the F_B population, the F_A population, which is enriched for ACTA2 persists in the remission state. The percentages of the F_A sub-population in active versus remission states were also similar. 

Increasing the resolution during clustering tree analysis failed to identify additional F_A subpopulations. On the other hand, increasing the resolution during cluster tree analysis led to the identification of more F_B subpopulations. Our preliminary analysis of the F_B sub-population suggests there may be shifts in the subsets of this group in active versus remission EoE. The evolution of the three F_B subclusters remains undefined. Pseudotime analysis represents a cell’s distance from its precursors in transcriptional space [24], and such an analysis of scRNA-seq in Barrett’s esophagus has identified links between fibroblast subpopulations with differentiation of the contractile and desmoplastic/ECM-depositing fibroblast phenotypes from the early immune cell class phenotype [15]. Future studies with larger cell numbers amenable to a pseudotime/trajectory type analysis may similarly identify a role for fibroblast differentiation or development in fibroblast subsets in EoE. 

Evaluation of genes enriched in F_A and F_B subpopulations shows that F_A is enriched for ACTA2, TAGLN, MYL9, and RGS5. Others have utilized ACTA2, TAGLN, and MYL9 as contractile myofibroblast markers [14,15,25,26], while RGS5 has also been referred to as a pericyte marker in scRNA-seq studies of the colon [14]. F_B was notably enriched for elastic fiber genes *FBLN1* and *FN1* [27] along with several COLs. SERPINE1 another fibrosis mediator was mostly enriched in F_A. Our work suggests that the transcriptomic contribution of each of these populations shifts in disease state. In the F_B population, there is an upregulation of epithelial–mesenchymal transition pathway in active EoE versus remission. In the F_A population, however, there is an overall downregulation of the TNF-α via the NFKβ pathway in active EoE versus remission EoE, suggesting a previously undescribed role for fibroblasts in the remission state. Previous studies have suggested an increase in the TNF-related cytokine, TNFSF14 or LIGHT secretion from a subset of fibroblasts from ulcerative colitis patients with active inflammation. A role for TNFα is also described in EoE. For example, LIGHT-expressing cells are present in the esophagus and increase in number in active EoE [16]. In addition, experimental studies using an esophageal epithelial cell line and primary normal fetal esophageal fibroblasts showed that epithelial cell-derived conditioned media stimulated fibroblast secretion of TNFα which in turn stimulated TGFβ secretion from epithelial cells as well as secretion of LOX, which has previously been shown to be upregulated in EoE [12] and which helps cross-link collagen molecules to form fibers [27]. 

Utilization of primary fibroblast cultures with differential *ACTA2* enrichment and modeling active disease with IL13 treatment generally validate DEGs identified in our analysis of the scRNA seq and support the findings of our analyses. More profound upregulation of *POSTN*, *TNC*, *LUM*, *FN1*, *COL6A1*, *COL6A2*, *COL6A3*, and *COL1A2* in *ACTA2−* cells treated with IL-13 is consistent with scRNA-seq DEG showing upregulation of these genes in F_B vs. F_A subpopulation in active EoE. Similarly, more profound upregulation of *RGS5* and *EGFL6* in *ACTA2+* primary cells is consistent with the upregulation of these genes in scRNA-seq analysis. 

Cell–cell communication analysis with CellChat also reiterated the importance of the fibroblast population in cross-talk with other cell populations. CellChat identifies disease-specific cell–cell communications based on a curated database of molecular interactions involving all known structurally defined ligand–receptor interactions. These interactions are divided into three categories: (1) interaction of secreted ligands and their specific binding receptors (secreted signaling interactions), (2) direct and indirect interaction of the extracellular matrix (ECM) and cell surface receptors (ECM–Receptor interactions), or (3) interaction of complementary proteins on the surfaces of neighboring cells (cell–cell contact interactions). Although proliferating epithelial cells contributed the most to the variation in cell–cell communication networks in the samples analyzed (total contribution score 8.32), this contribution was closely followed by the F_B (total contribution score 8.25) and F_A (total contribution score 8.24) fibroblast subpopulations. 

CellChat identified the inflammatory chemokine CXCL12 and amyloid precursor protein APP as F_B fibroblast ligands in secreted and cell–cell contact signaling with lymphocytes, epithelial, and endothelial cells. CellChat also identified COL1A2, COL6A1, COL6A2, COL6A3, and COL6A5 as F_B and F_A fibroblast ligands in ECM–receptor interactions with epithelial cells. F2R or PAR-1 is a protease-activated thrombin receptor that is present in multiple cell types and tissues with upregulation reported across a wide range of conditions. F2R expression is upregulated in smooth muscle actin-positive breast fibroblasts surrounding carcinoma cells [28] and in activated fibroblasts in arteries and veins [29] An increase in F2R has been reported in various GI tumors including cholangiocarcinoma, colon adenocarcinoma, esophageal carcinoma, rectum adenocarcinoma, and in stomach adenocarcinoma, where higher F2R expression was associated with infiltration of a number of immune cell populations including CD4+ lymphocytes, macrophages, and eosinophils. F2R silencing via siRNA transfection studies demonstrated that F2R promoted stomach adenocarcinoma cell proliferation, migration, and invasion [30]. Interestingly, our analysis identified an upregulation in mast cell to fibroblast (CTSG_F2R) and lymphocyte to fibroblast (PRSS3-F2R) signaling in active vs. remission EoE. Along with eosinophils, these are the immune cell populations implicated in EoE pathogenesis [31]. Future work will include small molecule inhibitor studies to complement these findings. 

Overall, our findings highlight a critical role of the fibroblast population in cell–cell communication in active and remission EoE, despite their overall low number in the entire dataset. Although it may be somewhat surprising that immune cells (myeloid and lymphocytes) do not have a larger contribution score prominence, the prominent role of epithelial cell populations per CellChat is in line with other publications questioning whether EoE is a primary inflammation-driven disease or rather an esophageal epithelial proliferative disorder [32]. Future work will be directed at the interrogation of identified ligand–receptor interactions with mutation or small molecular studies.

A limitation of this study is our inability to perform additional analysis of the fibroblast population in normal biopsies included in this publicly available dataset. Further rigorous analysis of F_B subsets was also limited by small numbers, although the suggestion of shifts in these subsets in active versus remission states merits further inquiry. Limitations of scRNA-seq relevant to our analysis include the possibility that gene detection could vary by cell or sample and it is possible that ACTA2 in a particular cell was not well detected but that the cell could still have all the other or many other markers that define its phenotype. As such, basing our results on high vs. low expression of this one gene could be biased given the known limitations scRNA-seq. To address this, we repeated our analyses with additional markers (*ADIRF*, *MYL9*, *COX412*, *FRZB*, *TINAGLI*, *CPE*) with similar results showing 2 broad populations of fibroblasts. 

Another potential limitation is that we utilized previously characterized normal ACTA2+ primary human esophageal myofibroblasts and commercially purchased primary human esophageal fibroblast cultures as models of the F_A and F_B sub-populations, respectively, based on enrichment of *ACTA2* expression. We have not yet evaluated the expression of other genes enriched in the F_A vs. F_B subpopulations, e.g., *TAGLN*, *MYL9* or examined proteins. We recognize that primary cultures derived from normal human esophagus most likely do not completely replicate the F_A and F_B sub-populations identified by our analysis of an integrated active and remission EoE dataset. Furthermore, a general limitation of the field is that classifications of heterogenous stromal cell populations have been heretofore based on evaluation of a few selected genes (e.g., CD90, SMA, vimentin), that have not been consistently utilized across all studies, potentially contributing to inconsistencies across naming of cell types across prior publications. Unbiased classification of fibroblast populations will be achieved with the advent of more widespread use of scRNA-seq. We also recognize that while treatment with IL-13 is a well-established and commonly utilized method of modeling active disease, it likely does not recapitulate the complex multi-cellular environment of EoE in vivo. Finally, we did not model remission EoE and while we appreciate this is an additional limitation of this study, we hope to use this work to further interrogate these populations in biopsies of EoE patients.

## 5. Conclusions

In conclusion, this proof-of-concept study adds to the literature demonstrating the complexity of the esophageal stroma in disease states such as EoE. Future studies, specifically focused on mesenchymal cells (non-epithelial, non-endothelial-, non-immune cells) including both normal and disease states (active and remission) with potentially greater numbers of fibroblasts available for analysis, are critical to identify populations that contribute to fibrostenotic and/or epithelial remodeling in EoE. A more refined understanding of the contribution of fibroblasts could provide insights into disease pathogenesis and fibrostenotic complications. Future studies are warranted to validate these findings in patients, to better characterize the fibroblast sub-populations and to define their interactions in different disease states. 

## Data Availability

The datasets used and/or analysed during the current study are available from the corresponding author on reasonable request. R scripts used for data analyses in our study can be found in the public GitHub repositories (Supplementary Table S2).

## Supplementary Materials

The following supporting information can be downloaded at: https://www.mdpi.com/article/10.3390/biology13070461/s1, Figure S1: ElbowPlots determining how many principal components are needed to capture the majority of the variation in 10 integrated datasets (A), 8 integrated datasets (B), and 8 integrated fibroblast sub-clustering datasets (C); Figure S2: Clustering tree showing how cells move as the clustering resolution is increased; Table S1: Primers used for qRT-PCR; Table S2: Summary of public GitHub repositories for R scripts used for data analysis; Table S3: Top 10 representative marker genes for each major cell type identified in the original scRNA analysis [18] are listed; Table S4: Top 10 representative marker genes for each epithelial sub-type identified in the original scRNA analysis [18]; Table S5: Top 10 representative marker genes for fibroblasts identified in the original scRNA analysis [18] and relative expression in F_B and F_A; Table S6: List of DEGs for fibroblast genes identified by CellChat.
