# Supplementary material for: Differential Contributions of Fibroblast Subpopulations to Intercellular Communication in Eosinophilic Esophagitis
Source: Biology (Basel). 2024 Jun 21;13(7):461. doi: 10.3390/biology13070461 (PMC11273487; doi:10.3390/biology13070461)
Supplement: Supplementary file 1 [file biology-13-00461-s001.zip › AS4_final_Supplementary Table 1_2_3_4_5_6.pdf]

Supplementary Table S1

Primers used in study for qRT-PCR

Gene Name	Primer (5'→3')
ACTA2	F: CTATGCCTCTGGACGCACAACT
	R: CAGATCCAGACGCATGATGGCA
POSTN	F: CAGCAAACCACCTTCACGGATC
	R: TTAAGGAGGCGCTGAACCATGC
LUM	F: AACATACCAACTGTCAATGAAAACC
	R: TGCCATCCAAACGCAAATGCTTG
RGS5	F: GAAGCCAGACTCAGTTGGTGAC
	R: GGAGTTTGTCCAGGGAATCACG
EGFL6	F: GATTCTGGTCCAAAGGAAAGCGC
	R: CTCGATCAGCAGGATTCCAGTC
CCL26	F: GGGAGTGACATATCCAAGACCTG
	R: CAGACTTTCTTGCCTCTTTTGGTA
TNC	F: ATGTCCTCCTGACAGCCGAGAA
	R: AGTCACGGTGAGGTTTTCCAGC
IL13RA1	F: CCTGAATGAGAGGATTTGTCTGC
	R: CAGTCACAGCAGACTCAGGATC
IL13RA2	F: GTGGAGTGATAAACAATGCTGGG
	R: TGGGTAGGTGTTTGGCTTACGC
FN1	F: ACAACACCGAGGTGACTGAGAC
	R: GGACACAACGATGCTTCCTGAG
COL6A1	F: GCCTTCCTGAAGAATGTCACCG
	R: TCCAGCAGGATGGTGATGTCAG
COL6A2	F: CGTGGAGACTCAGGACAGCCA
	R: CCTTTCAAGCCAAAGTCGCCTC
COL6A3	F: CCTGGTGTAACTGATGCTGCCA
	R: AAGATGGCGTCCACCTTGGACT
COL1A2	F: CCTGGTGCTAAAGGAGAAAGAGG
	R: ATCACCACGACTTCCAGCAGGA

Supplementary Table S2

Summary of public GitHub repositories for R scripts used for data analysis

Name of the Repository	R packages	URL
satijalab/seurat	Seurat	https://github.com/satijalab/seurat
sqjin/CellChat	CellChat	https://github.com/sqjin/CellChat
ctlab/fgsea	fgsea	https://github.com/ctlab/fgsea
cole-trapnell-lab/monocle3	Monocle 3	https://github.com/cole-trapnell-lab/monocle3

Supplementary Table S3

**Epithelial cells**		**Lymphocytes**		**Myeloid cells**		**Mast cells**		**Endothelial cells**	
gene	log2FC	gene	log2FC	gene	log2FC	gene	log2FC	gene	log2FC
S100A2	1.28	GNLY	2.25	HLA-DRA	2.39	TPSAB1	2.39	PLVAP	1.80
SPRR2D	1.18	CD7	1.99	HLA-DPB1	2.30	CPA3	2.31	RAMP2	1.76
CSTA	1.18	CCL5	1.97	HLA-DPA1	2.19	HPGDS	2.19	AQP1	1.76
CNFN	1.16	KLRB1	1.93	HLA-DQA1	1.96	LTC4S	1.96	ENG	1.47
SPRR2A	1.10	NKG7	1.85	HLA-DQB1	1.87	CTSG	1.87	EMCN	1.43
SPRR3	1.09	GZMA	1.36	LYZ	1.78	VWA5A	1.78	ECSCR	1.36
S100A8	1.05	CD3D	1.28	AIF1	1.75	KRT1	1.75	CLDN5	1.33
FAM25A	0.96	IFNG	1.16	C1QA	1.44	SLC18A2	1.44	IFI27	1.22
CSTB	0.95	CD3E	0.95	C1QB	1.42	NSMCE1	1.42	INSR	1.12
CRCT1	0.91	CD2	0.83	C1QC	1.39	RGS13	1.38	DARC	1.11

Top 10 representative marker genes for each cell type identified in Rochman et al are listed. The average log2FC determined by our analysis of the publicly available data is shown. Adjusted P values for all genes are 0.

Supplementary Table S4

Quiescent		Proliferating		Trans1		Trans2		Differentiated low		Differentiated high	
gene	log2FC	gene	log2FC	gene	log2FC	gene	log2FC	gene	log2FC	gene	log2FC
DST	1.24	HMGB2	1.22	DSC2	0.80	SERPINB3	0.50	TGM3	1.18	RNASE7	1.47
KRT15	1.08	STMN1	1.20	DSG3	0.62	ACTB	0.33	FAM3D	1.16	HILPDA	1.39
GPNMB	0.97	H2AFZ	1.09	DSP	0.57	SH3BGRL3	0.27	CLCA4	1.02	MT1G	1.28
IL1R2	0.73	TUBA1B	1.08	PKP1	0.57	CRABP2	0.27	PIM1	0.94	KRT16	0.87
TSLP	0.70	TUBB	1.04	TRIM29	0.44	CST3	0.26	AIM1	0.87	DKK1	0.81
WNT10A	0.67	HIST1H4C	1.02	CDH1	0.38	CSTA	0.26	MLLT4	0.84	MT1H	0.69
DLK2	0.66	RRM2	0.96	DHCR24	0.34	HSPB1	0.33	WDR26	0.83	MT2A	0.91
GLTSCR2	0.67	UBE2C	0.95	MAST4	0.32	MSMO1	0.27	PADI1	0.79	MT1E	0.84
ZFP36L2	0.66	CCNB1	0.86	ELL2	0.31	SERPINB4	0.52	DIO2	0.74	KRTAP3-2	0.29
TXNIP	0.58	TOP2A	0.86	GJA1	0.30	PRDX5	0.25	CTSV	0.70	SPRR2D	0.55

Top 10 representative marker genes for each epithelial sub-type identified in Rochman et al are listed. The average log2FC determined by our analysis of the publicly available data is shown. Adjusted P values for all genes are 0.

Supplementary Table S5

Fibroblasts			
Gene	Log2FC	Log2FC F_B vs F_A	adj p-value F_B vs F_A
LUM	2.36	1.93	2.56e-91
DCN	2.29	0.653	2.642e-56
COL3A1	2.10	1.073	7.39e-58
PTGDS	2.06	2.023	2.42e-64
APOD	2.04	1.99	9.55e-79
COL1A2	2.01	0.86	1.64e-49
MFAP4	1.94	1.68	1.57e-71
C1S	1.88	0.74	1.34e-36
RGS5	1.85	-1.76	7.78e-85
FBLN1	1.85	1.87	8.67e-79

Top 10 representative marker genes for fibroblasts identified in Rochman et al are listed. The average log2FC determined by our analysis of the publicly available data is shown in the column labeled Log2FC (adjusted P values for all genes are 0). In addition, the average log2FC for expression comparison in F_B vs F_A subpopulation is shown. This data is also reflected in the heat map in Figure 2. Positive and negative values represent upregulation and downregulation in F_B vs F_A, respectively.

Supplementary Table S6

	DEG (log2FC/adjusted p-value)			
Gene	F_A vs F_B in active	F_A vs F_B in remission	F_A: active vs remission	F_B: active vs remission
CTSG	-	-	-	-
CXCL12	-	0.91/5.66e-15	-	-
F2R	-	-	-	-
APP	-	-	-	-
COL1A2	1.70/2.06e-18	0.93/3.03e-29	-	-
COL6A1	1.12/2.49e-22	1.12/1.97e-32	-	-
COL6A2	0.96/4.39e-23	1.18/1.51e-36	-	-
COL6A3	1.26/3.65e-19	1.21/1.04e-26	-	-
COL6A5	1.23/7.80e-16	0.86/1.28e-14	-	0.69/3.49e-12

List of DEGs for fibroblast genes identified by CellChat; DEGs were defined as log2FC > 0.6 and adjusted p-value < 0.05. DEGs are shown for multiple comparisons. Positive and negative values represent upregulation and downregulation in F_B vs F_A in active or remission, respectively. Positive value represents upregulation in active vs remission in F_B. – represents non-DEG in the comparison.
