# Supplementary figures and images for: Differential Contributions of Fibroblast Subpopulations to Intercellular Communication in Eosinophilic Esophagitis
Source: Biology (Basel). 2024 Jun 21;13(7):461. doi: 10.3390/biology13070461 (PMC11273487; doi:10.3390/biology13070461)

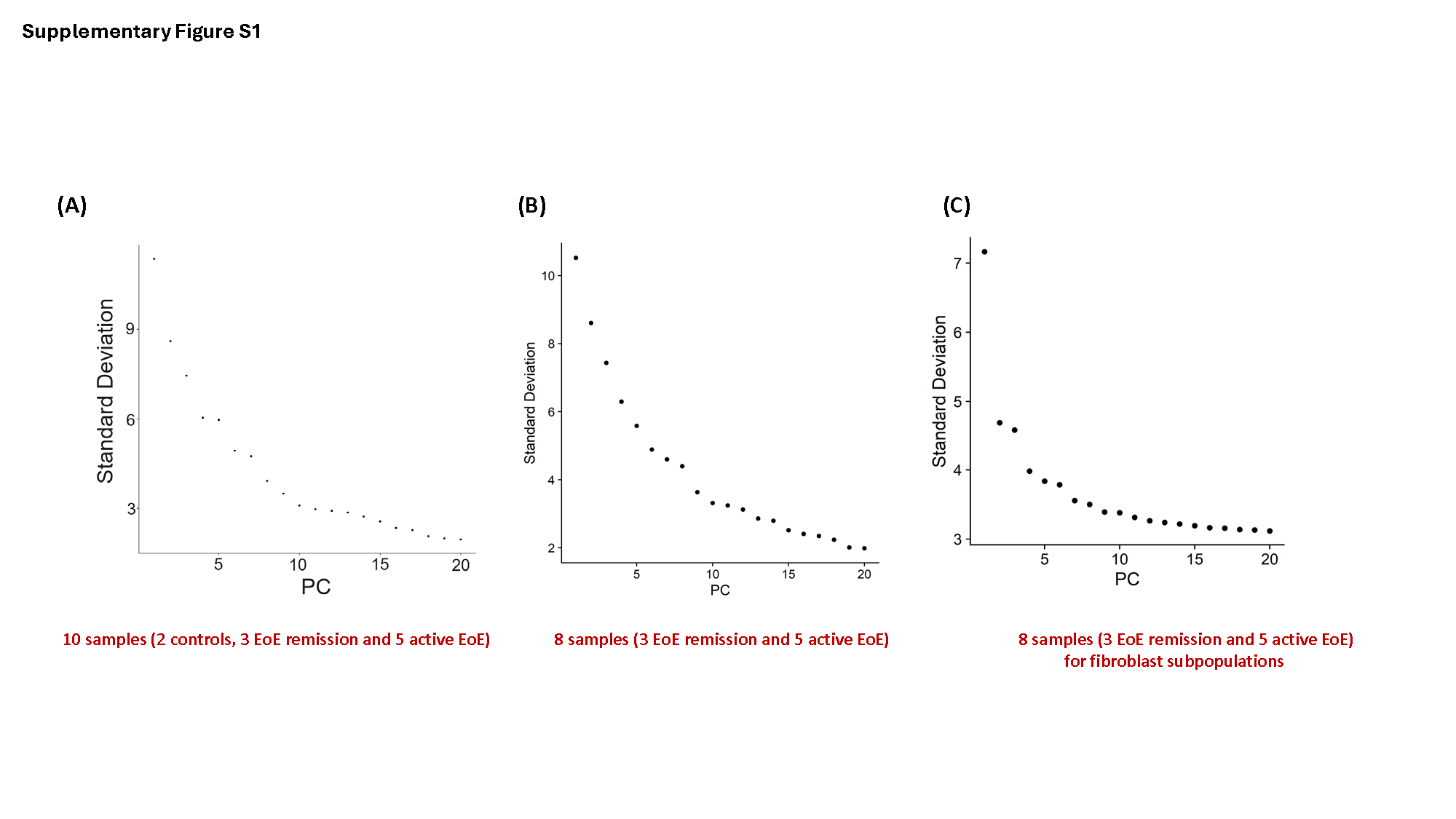

Supplement: Supplementary file 1 [file biology-13-00461-s001.zip › S1.tiff]

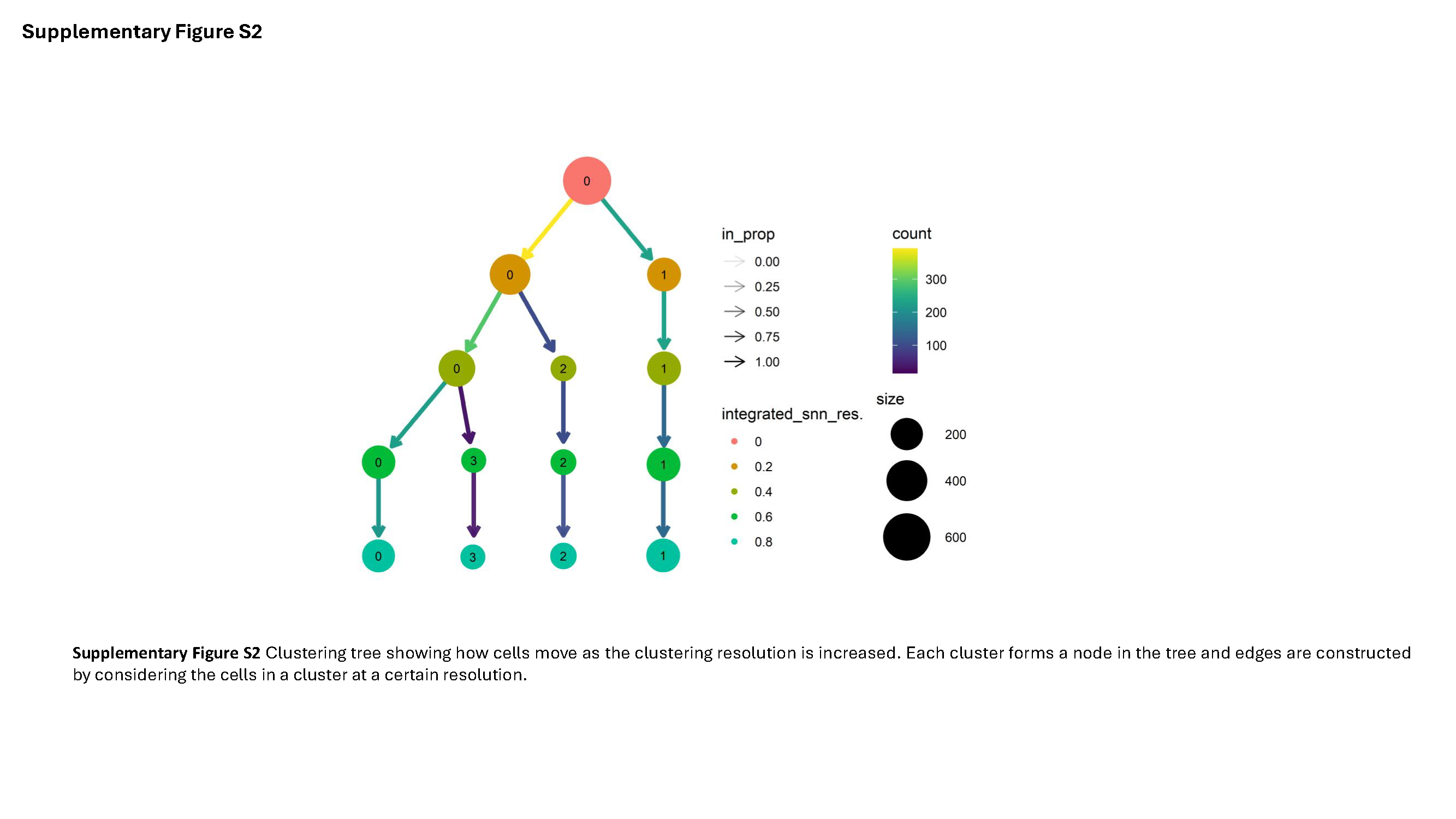

Supplement: Supplementary file 1 [file biology-13-00461-s001.zip › S2.tiff]
